# 

**Published:** 1869-02

**Authors:** 


### CONTENTS.

PAGE.

Case of Uterine Polypus, by Prof. George W. Hall, M. D ..... 93
Poisoning by Strychnia, by Prof. A. M. Carpenter, M. D ..... 94
Removal of Vessical Calculi, the nucleus of which was a pistol bullet, by Prof. J. C. Hughes, M. D ..... 98
Indolent Ulcer successfully treated by Compression, by J. F. Grimes, M. D., Wapello, Iowa ..... 99
Strangulated Hernia, by J. W. Hall, Toulon, Illinois ..... 100
SELECTIONS:
- Nature and Use of Carbolic Acid ..... 101
- Treatment of General Dropsy by the Hot Bath ..... 107
- Twisting Ends of Arteries ..... 108
- Preservation of Iodide of Iron ..... 108
- Treatment of Night Sweats in Consumption ..... 109
- Croup treated by Sulphur ..... 109
- How to Prevent Iodine from Staining ..... 109
- New Treatment for Tape Worm ..... 109
- Sulphurous Acid as applied to Wounds and Sores ..... 109
- Hydrophobia ..... 109
- Cure of Sickness attending Pregnancy by Bromide of Potassium ..... 110
- Open Cancer ..... 110
- List of Graduates of the College of Physicians and Surgeons, Keokuk, session of 1867—8 ..... 111

Reviews and Notices of Books, &c ..... 111
- Orthopædic Apparatus, by D. W. Kolbe ..... 113
- Local Anæsthesia and Atomization of Liquids ..... 114
- New Instrument, by Prof. John T. Hodgin ..... 114
- Dr. Teal's Invalid Bed Attachment ..... 114
- Robert Fulton Clokey in relation to the Irish Poor Law ..... 114

Exchanges ..... 115
EDITORIAL:
- The University and its Medical Department ..... 116
- Iowa Medical Journal ..... 117
- Our Medical College ..... 117
- Iowa State Medical Society ..... 118
- L. C. Dobyns ..... 118
- Dr. John F. Sanford ..... 118

OBITUARY:
- Dr. Edward Whinery, Dr. John P. Fleming, Dr. Cousins and Prof. Joseph N. McDowell ..... 119

